# Girls exhibit greater empathy than boys following a minor accident

**DOI:** 10.1038/s41598-021-87214-x

**Published:** 2021-04-12

**Authors:** Joyce F. Benenson, Evelyne Gauthier, Henry Markovits

**Affiliations:** Département de Psychologie, Université du Québec à Montréal, Montréal, H3C 3P8 Canada

**Keywords:** Evolution, Neuroscience, Psychology

## Abstract

Hundreds of studies find that girls and women report feeling greater empathy than boys and men in response to adverse events befalling others. Despite this, few non-self-report measures demonstrate similar sex differences. This produces the oft-cited conclusion that to conform to societal expectations of appropriate sex-typed behavior females report higher levels of empathy. Several studies of sex differences in areas of brain activation and on infants’ and young children’s behavior however provide suggestive findings that self-reports reflect actual underlying sex differences in experiencing concern about others. We demonstrate using behavioral indices that females experience more empathy than males after witnessing an adverse event befall a same-sex classmate. In our study, one member of a pair experienced a minor accident on the way to constructing a tower while a bystander observed. We measured whether bystanders ceased their ongoing activity, looked at the victim, waited for the victim to recover from the accident, and actively intervened to help the victim. Female more than male bystanders engaged in these activities. These behavioral results suggest that an adverse event produces different subjective experiences in females than males that motivate objectively different behaviors, consistent with findings from self-report measures of empathy.

## Introduction

The idea that women experience more empathy than men for others’ plights stems largely from knowledge of mammalian females’ typically greater responsibility for the care of offspring^1,2^. In humans, from early childhood through old age, females universally take more immediate responsibility than males for children’s and others’ survival^3–5^. A stronger response to threats to survival to genetic kin would enhance a mother’s reproductive success, thereby conferring greater genetic fitness on more versus less empathic mothers. Theoretically then, negative events that befall genetic kin should elicit stronger reactions in females than males. In contrast, mammalian males including humans often derive greater genetic fitness benefits from inseminating more females as opposed to investing in protecting the lives of fewer kin^2^. In men, this is evident in males’ universally greater desire for more sexual partners^6^.

If this theoretical framework is extrapolated to non-kin, then females should experience greater empathy than males at least to those with whom they have relationships. In support of this sex difference, several large studies demonstrate that when an adverse event befell a family member, a social network member, or a patient, both female family members and female professionals reported experiencing more frequent and severe distress than their male counterparts^7,8^. More generally, meta-analyses provide persuasive evidence that human females subjectively report more empathic concern than males for others, based on diverse measures of empathy across varied contexts, with a large effect size of *d* = 0.91^9^. As examples, in response to stories or films of stressful events befalling a protagonist, female children and adolescents subjectively report much higher sharing of negative emotions or empathy than their male counterparts do^10^. Likewise, by adulthood, women consistently score higher than men on the general Empathy Quotient (EQ) scale which consists of self-reports of one’s own caring for and knowledge about interactions with other individuals^11^.

In marked contrast to the compelling theoretical framework and consistent self-reports of females’ greater experiences of empathy, sex differences in non-self-reports of empathy typically are not found^10,12–14^. Decades of research during the twentieth century underscore the conclusion that sex differences in empathy arise almost exclusively from self-report measures. This surprising discrepancy therefore invites further investigation.

Empathy constitutes a complex construct that consists of a range of definitions and measures including affective reactions such as emotional matching, and cognitive processes such as perspective-taking^15,16^. The sex difference in empathy that is reported subjectively pertains primarily to experiencing emotional concern, including sharing an individual’s painful emotion, sympathy for another’s plight, and experiencing vicarious personal distress^10,13,14,17,18^.

Because few behavioral or physiological responses yield sex differences in empathy^13,19^, researchers often address the disparity between sex differences in self-reports versus objective measures by concluding that universal sex-typed expectations pressure females into reporting greater empathy than males^10,20,21^. This interpretation is supported by findings that when demand characteristics supporting greater expressions of empathy increase, females demonstrate correspondingly higher empathy^10^. Consistent with this, a recent study with over 10,000 participants viewing animations of people being accidentally or intentionally caused pain concluded that sex differences in empathy were virtually all due to sex-stereotyped norms and expectations for women to be perceived as more caring and nurturant^22^.

There are two areas however in which objective measures are consistent with subjective reports of females’ stronger feelings of empathy in response to others’ adverse events. First, several specific brain regions associated with empathy often are found to be larger or more highly activated in response to viewing adverse events in females than males. For example, in both sexes, higher self-reported empathy was associated with larger brain areas in the human mirror neuron system (MNS), an area associated with matching others’ emotions and actions^23,24^. Furthermore, young adult females had larger gray matter volume compared with males in a number of brain regions associated with the human MNS^25^. Other brain imaging studies also have found linkages between various self-reported empathy scales and several regional gray matter brain volumes associated with empathy including the MNS, although there is a lack of consistency across studies^26,27^.

Second, several behavioral studies with infants and young children with real life victims provide objective evidence that females experience greater empathy than males. Studies with children are especially valuable for countering the interpretation that sex differences are due solely to conformity to societal expectations, because socialization pressure to adhere to sex-typed norms has had less time to exert its influence. Thus, in several studies female newborns cry more than males to a female newborn’s cry, although no sex differences exist in response to a male newborn’s cry^16^. Further, when 14- and 20-month-old infants witnessed a nearby female adult (a mother or female experimenter) simulating getting her hand trapped in a suitcase or hurting her knee, female infants were more likely than males to express verbally or non-verbally their concern and or negative emotions, attempt to figure out what happened, or exhibit prosocial behavior as rated by trained coders, whereas boys were more likely to not respond at all^28^. Likewise, 4–5 year-old children at risk of developing behavior disorders were exposed to a female experimenter and their mothers simulating hurting one of their feet^29^. Concern (empathy, sympathy, and helpfulness) versus disregard (anger, amusement, and avoidance of victim) were rated. Girls exhibited greater concern for others, whereas boys displayed greater disregard.

These behavioral studies of empathy are unusual because they include live expressions of distress by a person in close proximity. It is possible however that across these studies girls exhibited greater concern than boys because the victims more often were females. As described, in response to infant crying, sex differences occur only when a female, but not a male, newborn cries^16^. More generally, other studies have found that greater concern typically is directed towards victims of the same sex^12,16^.

In perhaps the most naturalistic study of empathy, Caplan and Hay (1989) examined 3- to 5-year-old children’s behavioral reactions to a victim’s spontaneous distress at a preschool. After identifying episodes of distress, they classified bystanders’ concerned responses into 3 categories: looking at the distressed child for at least 3 s, ceasing their own ongoing activity, and actively intervening. Unfortunately, sex differences were not examined and several bystanders, including the teacher, were present which likely modified any individual child’s response. Nonetheless, the categorization scheme is valuable for operationalizing spontaneous behaviors demonstrating empathy in a naturalistic study. Furthermore, the classification of behaviors into looking, ceasing activity, and active intervention can be objectively measured without resorting to subjective impressions that can be biased by societal expectations.

Because behavioral studies of sex differences in response to real life adverse events are rare, the goal of the current study was to combine elements of prior behavioral studies to test whether girls would exhibit greater concern than boys using objective measures. Therefore, we engineered a standardized, commonplace accident that occurred in a context in which the only witness to the accident was a familiar same-sex child, so no adult was present who could be expected to provide assistance. Because universally children segregate themselves by sex^30,31^ and individuals are believed to exhibit more sympathy for victims of their own sex at least in childhood^10,16^, only same-sex children were included. We selected 5- to 7-year-old children, because they were old enough to cope with a minor accident by themselves, due to qualitative advances in physical, emotional, and cognitive independence at this age^32,33^. Yet, they are still children and hence less subjected to societal expectations for conformity to sex roles than adults are.

Our procedure consisted of bringing two same-sex classmates to a teacher’s room devoid of objects interesting to children and providing an attractive task for them to complete. The task consisted of constructing a tower from small plastic blocks on a table that was situated at the far end of a room. In order to work on the tower, children had to carry the blocks in a basket from the starting position near the door to the table at the far end of the room. On the way to the table, the victim’s basket of blocks broke, scattering the blocks on the floor. From the videotapes of the accident, based on Caplan and Hay’s (1989) coding scheme, we coded whether the bystander looked at the distressed child for at least 3 s, ceased their own ongoing activity for at least 1 s, ceased ongoing activity by waiting until the victim had a chance to begin building their own tower before placing their own blocks, and actively intervened by picking up the victim’s block or basket. Our primary hypothesis was that girls would demonstrate more empathy than boys as assessed by these four measures.

We also created two conditions- one in which the bystander and victim worked on a joint tower and a second in which they worked separately. Collaboration on a joint task has been shown to increase egalitarian distribution of rewards in young children relative to separate conditions^34^. Extrapolating from this, we surmised that collaboration would induce stronger feelings of connectedness which would enhance feelings of empathy in response to the breaking basket. Thus, our second hypothesis was that sex differences would be accentuated when the children were working on a joint task.

## Results

Each child could engage in 0–4 empathic behaviors. 7 girls and 5 boys did not engage in any empathic behavior, whereas 8 girls and 0 boys engaged in all 4 behaviors. Table 1 presents the numbers and percentages of female and male bystanders who engaged in each of the four empathic behaviors listed in order of total frequency of occurrence (see Table 1).

**Table 1:** Number of female and male bystanders who engaged in each measure of empathy in order of frequency of occurrence.

Measure of Empathy	Females (n = 32)		#	Males (n = 23)		*p*
Condition	#	%		%	χ^2^	
**Ceased ongoing activity ≥ 1 s**	25	78.1	18	78.3	.00	.990
Joint^a^	13	84.6	11	72.2		
Separate	12	85.7	7	70.0		
**Looked at victim ≥ 3 s**	22	68.8	9	39.1	4.77	.029
Joint	11	61.1	5	38.5		
Separate	11	78.6	4	40.0		
**Ceased ongoing activity until victim placed a block on the tower**	11	34.4	1	4.3	7.07	.008
Joint	7	38.9	0	0.0		
Separate	4	28.6	1	10.0		
**Actively intervened by picking up victim’s block or basket**	11	34.4	4	17.4	1.95	.163
Joint	9	50.0	2	15.4		
Separate	2	14.3	2	20.0		
**No empathic behavior**	7	21.9	5	21.7	.00	.990
Joint	5	35.7	2	15.4		
Separate	2	14.3	3	30.0		
**All 4 empathic behaviors**	9	28.1	0	0.0	7.73	.005
Joint	7	50.0	0	0.0		
Separate	2	14.3	0	0.0		

^a^18 female and 13 male pairs participated in the joint condition; 14 female and 10 male pairs participated in the separate condition. Cell counts were too low for statistical comparisons within conditions.

As depicted in Table 1, for the individual measures, chi-square analyses showed that following the accident, significantly more girls than boys looked at the victim for ≥ 3 s and ceased their ongoing activity until the victim recovered sufficiently to place his/her first block. Additionally, significantly more girls than boys engaged in all four empathic behaviors.

An overall measure of bystander’s empathy then was created using a hierarchically weighted score for each of the empathic behaviors according to the degree of effort it required (and its frequency of occurrence). Ceasing ongoing activity for ≥ 1 s was given a score of 1; Looking at the victim for ≥ 3 s a 2; Ceasing ongoing activity until the victim could begin building a 3; and Actively intervening a 4. The overall score for empathy for each bystander consisted of the sum of the four scores and served as the dependent variable.

A loglinear General Linear Mixed Model (GLMM) was conducted on overall empathy with sex, condition, age, and their two-way interactions as fixed factors, and school as a random effect. Consistent with the primary hypothesis, the effect of sex was significant, *F* (1, 48) = 6.98, *p* = 0.011 [β = − 3.38, CI = − 6.14 to − 0.61] with females exhibiting greater empathy than males. Support for the secondary hypothesis however was not conclusive as the Sex X Condition interaction did not attain conventional significance levels, *F* (1, 48) = 3.22, *p* = 0.079. A significant effect of sex X age, *F* (1, 48) = 5.61, *p* = 0.022, was found but follow-up regressions between overall empathy and age were not significant within each sex.

Because both ceasing ongoing activity until the victim could begin building and active intervention required ceasing ongoing activity for ≥ 1 s, empathy scores could be improperly inflated. To ensure that each of the behaviors was independent therefore, we constructed a second overall score of empathy by omitting ceasing ongoing activity for ≥ 1 s. The second overall empathy measure consisted only of the weighted scores for 3 measures: looking at the victim ≥ 3 s, ceasing ongoing activity until the victim could begin building; and actively intervening. The second overall score for empathy for each bystander consisted of the sum of the three scores and served as the dependent variable in a GLMM with the same factors as before. Consistent with the primary hypothesis, results again showed that the effect of sex was highly significant, *F* (1, 48) = 10.58, *p* = 0.002 [− β = 5.05, CI = − 8.41 to − 1.67] with females exhibiting greater empathy than males did. The secondary hypothesis that the sex difference would be accentuated in the joint condition was significant, Sex X Condition, *F* (1, 48) = 5.95, *p* = 0.018 [β = − 1.03, CI = − 1.87 to − 0.18]. The sex difference was greater in the joint than the separate condition. Table 2 displays the raw means for female and male bystanders by condition for both overall weighted empathy measures (see Table 2). A significant effect of sex X age, *F* (1, 48) = 8.25, *p* = 0.006, again was found but again separate follow-up regressions were not significant within sex.

**Table 2:** Raw means (and SE) of two overall weighted empathy measures for female and male bystanders by condition.

Condition	Females			Males		
	M	(SE)	n	M	(SE)	n
**Empathy Score (4 measures)**						
Joint	5.17	(1.03)	18	2.69	(.55)	13
Separate	3.93	(.82)	14	2.90	(.89)	10
Combined	4.63	(.68)	32	2.78	(.48)	23
**Empathy Score (3 measures)**						
Joint	4.39	(.99)	18	1.39	(.53)	13
Separate	3.00	(.79)	14	1.90	(.85)	10
Combined	3.78	(.66)	32	1.61	(.47)	23

**Figure 1:**
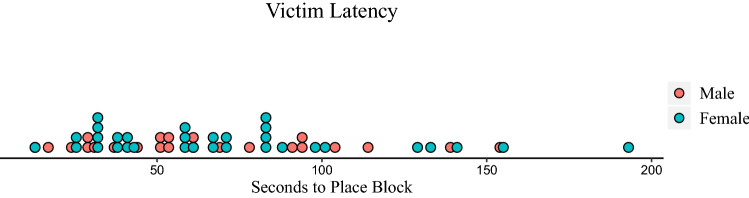
Latency (seconds) following the accident to begin building the tower for female and male victims.

The sex difference in empathy could be due however to differences in how long female versus male victims took to recover from the accident. To examine this possibility, we tabulated the number of seconds that elapsed between the accident and the victim’s placing his/her first block on the tower (after collecting their fallen blocks and bringing them to the table). Figure 1 displays the victims’ latencies following the accident to place their first blocks (see Fig. 1). We entered this latency measure as the dependent variable in a linear GLMM analysis with a log transformation and sex, condition, age and their 2-way interactions as fixed factors, and school as a random effect. No effect of sex was found, *F* (1, 48) = 0.59, *p* = 0.45, nor were any other effects significant.

**Figure 2:**
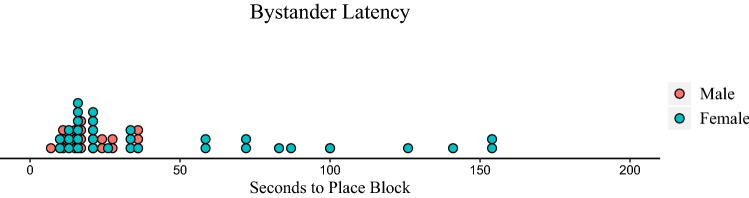
Latency (seconds) following the accident to begin building the tower for female and male bystanders.

As a final illustration of the impact of the accident on females versus males, we measured the latency for bystanders to place their first blocks on the tower following the victim’s accident by following the same procedure as for the victim. Figure 2 displays the bystander’s latencies following the accident to place their first blocks (see Fig. 2). Because the distribution was positively skewed, we entered the bystander’s latency to begin working on the tower as the dependent variable in a GLMM analysis using an inverse gaussian distribution again with sex, condition, and their interactions as fixed factors and school as a random effect. The effect of sex was significant, *F* (1, 48) = 6.55, *p* = 0.014 [β = − 112.60, CI = − 207.12 to − 18.07]. Consistent with the finding that females exhibit more empathic behaviors than males, female bystanders took significantly longer than males to begin working on their own towers. The sex X condition interaction however did not attain significance, *F* (1, 48) = 2.82, *p* = 0.10. Additionally, there was a significant effect of age, *F* (1, 48) = 15.28, *p* < 0.001 [β = − 25.00, CI = − 38.58 to − 11.41], showing that younger bystanders took longer than older bystanders to place their first blocks, and a significant sex X age interaction, *F* (1, 48) = 5.83, *p* = 0.02, [β = 16.32, CI = 2.73–29.91]. Again, separate follow-up regressions between latency and age were not significant within sex. No other effects were significant.

## Discussion

Following a minor accident, 5- to 7-year-old female bystanders exhibited more empathic behavior than male bystanders. These behavioral findings, along with results from studies of brain activation^26,27^ and from behavioral studies with infants and young children^12,16^, support the ubiquitous findings that human females report experiencing greater empathy than males at all ages^9^. This suggests that even though facial expressions and many physiological assessments rarely yield sex differences in empathic responsivity^10,13,18^, it is highly likely that females do experience greater feelings of empathy which motivate them to behave in ways that display more concern for victims. Behavioral studies with real life, proximate victims and brain activation measures may be best able to identify sex differences in empathy as others have proposed^1,16^.

Further, it appeared that the joint condition accentuated the sex difference, though the effect only attained significance in the overall empathy score with 3 measures. The empathy score with the 4 measures was only marginally significant. Nevertheless, it seems reasonable that a condition that increases the proximity and dependence between individuals would augment human females’ investment in one another.

This study builds on behavioral studies of bystanders who witnessed a distressing event that were conducted with newborns, infants, and young children all of which provided evidence that females do experience greater empathy than males^10,13,16^. This study extends that finding to middle childhood using the measures developed by Caplan and Hay to study concern in older children in a naturalistic setting^35^. Contrary to most prior studies however, the current study included victims who were always of the same sex and who were familiar because they were classmates. Furthermore, the accident was naturalistic yet standardized, unlike several prior behavioral studies in which the accident was staged. Additionally, no external observers, particularly adults, were present, thereby reducing demand characteristics pressuring females to behave in stereotyped ways or providing authority figures who could be perceived as better able to respond to a victim’s distress. Finally, empathic concern as measured through behavior that is costly to the bystander in terms of lost time and effort provides greater validity than measures such as self-report or facial expressions.

Theoretically, females’ greater concern regarding a stressor is consistent with predictions from parental investment theory that mammalian females exhibit greater responsiveness than males to threats to their offspring^2,5^. Findings also resemble sex differences found in self-reports about real life contexts in which others with whom participants have close relationships experience serious adverse events^7,8,36^. Results from the current study suggest that even a classmate who is not a close friend who experiences a minor accident elicits sex differences in empathy.

Our findings suggest when an adverse event occurs, more females than males will be affected. This is consistent with studies showing that more women than men are concerned about hardships that befall family members, individuals in their social networks, and those for whom they are professionally responsible^7,8^. More females than males also would be expected to offer supportive responses to victims, entailing greater disruptions to their own lives.

Limits of the study include the relatively small number of dyads and the utilization of only a single task. Further, because no sharing of resources occurred in the current study, the expected impact of working on a joint task found in prior studies of reward distributions may have been somewhat attenuated^34^. Inclusion of more participants from a wider age range, and replication with a new task would enhance the study’s validity. Finally, it is possible that boys experience empathy in a way that has not been measured. In this study, most boys did cease their ongoing activity following the accident, but they engaged in few other empathic activities that we could measure.

In summary, after observing their partner’s accident, female bystanders demonstrated greater empathy than males. In contrast to the relatively consistent conclusion that no sex differences occur in empathy other than in self-reports, results suggest that it is possible to behaviorally observe sex differences in empathy using established measures.

## Method

### Participants

Kindergarten and first grade children from 3 schools serving a predominantly lower to middle class population were semi-randomly paired with another same-sex classmate, producing 32 female dyads, *M*_age years_ = 6.02 (*SE* = 0.13) and 23 male dyads, *M*_age years_ = 6.16 (*SE* = 0.15) in Montreal, Canada. The study was approved by the Université du Québec à Montréal ethics review board. All methods were carried out in accordance with the university’s ethical guidelines and regulations. Only children who received informed consent from their parents/guardians and who assented themselves to participate were included. To ensure that the sample was not biased due to strong bonds or enmity between the children, after we randomly paired children, we asked teachers to ensure that the selected pairs did not consist of close friends or children who did not get along. We revised any pairs who met either of these two criteria. The general procedure, including videotaping, was explained to all children before the study began.

### Procedure

**Figure 3:**
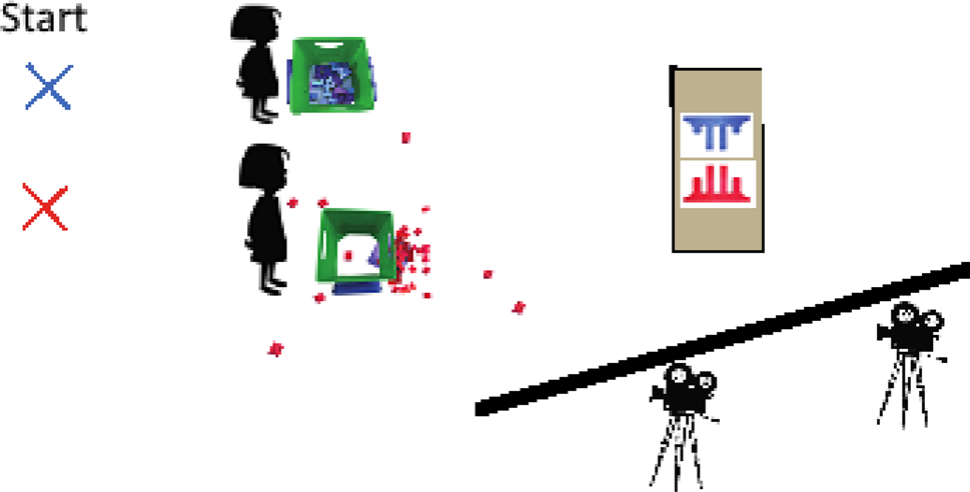
Layout of experimental set-up with images for the separate condition.

A male or female experimenter entered each classroom and invited two same-sex children to play a game that involved building a tower. Interested pairs were escorted to another room in the school that included a rectangular table at the far end and two baskets on the floor near the entrance. One basket contained red and the other contained blue Mega Bloks. A curtain positioned near the table occluded the video cameras and the experimenters. Figure 3 displays the experimental set-up (see Fig. 3). Videotaping began after the children entered the room.

Upon arrival, children were randomly assigned to sit behind the basket containing either the blue or red blocks. A female experimenter then told a story constructed through pilot testing to be equally attractive to girls and boys by including elements of both family and extraterrestrial life^37^. The experimenter explained that two extraterrestrial children had become separated from their parents who lived on another planet. To contact their parents, the extraterrestrial children needed to build a tower on which they could mount their special antenna to contact their parents. The children then were asked if they would be willing to help the extraterrestrial children by building the towers on which the extraterrestrial children could mount the antennae.

To construct the towers, children had to carry the basket in front of them filled with blue or red blocks to the table. A female or male experimenter then demonstrated the procedure to each child. The experimenter carried the child’s basket from the starting position on the floor to the table, then showed the child a computerized image of the tower to copy and the base on which to build the tower. The experimenter then removed a few blocks from the basket and showed the child how to place them on the base to build the tower according to the computerized image. The task was designed to be enjoyable and simple, and all children easily understood the instructions and agreed to participate.

**Figure 4:**
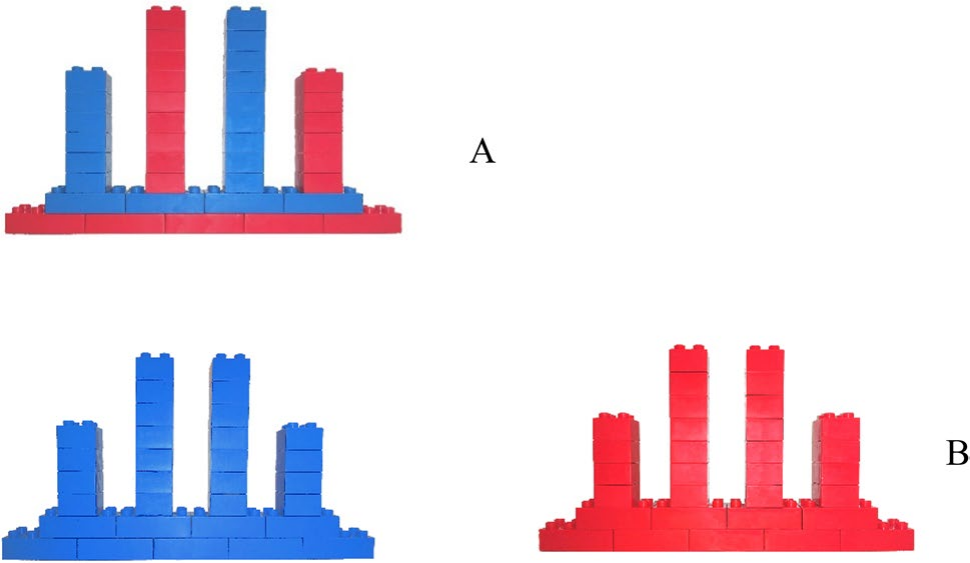
Tower to be constructed in the (**A**) joint condition and (**B**) separate conditions.

Each pair was randomly assigned to one of two conditions: joint or separate. In the joint condition, the pair copied a single image of a tower of alternating blue and red blocks. In the separate condition, each child copied their own image of a (blue or red) tower made only of their own (blue or red) blocks. In both conditions, the bystander’s image and base were in the identical position at one end of the table. In the separate condition, the accident victim’s image and base were at the opposite end of the table. Because two children worked on the joint tower, each had half as much work as children who worked on their own separate towers. The size of the tower was not doubled in the joint condition so that the perceptual affordances of the images, the bases, and the towers, were identical across conditions. The completed towers are displayed in Fig. 4 for the joint and separate conditions (see Fig. 4).

Before leaving the demonstration, children were told that after their tower was finished, they each would receive a surprise (a sticker) to thank them for their efforts to help the extraterrestrial children, but thank you gifts would not be provided until both children had finished building their tower(s). The experimenter then emphasized that there was no hurry, so children could take as much time as they needed to finish their tower(s).

The experimenters then led the children back to the starting position and asked each child to sit in their original positions in front of which the experimenter placed either the basket of blue or red blocks. One of the experimenters then left, and the remaining experimenter asked if the children were ready to begin. After both children assented, the experimenter mentioned that one of the baskets was somewhat broken. If it broke during the game, which it sometimes did, the experimenter stressed that it was not the children’s fault. However, the adults were going to be too busy to fix it right away. Therefore, the children should just pick up the blocks with their hands, bring them to the table, and build their towers.

The experimenter explained that both experimenters were going to be behind the curtain busily working, and neither could be disturbed. The experimenter then instructed the children to sit behind their baskets until both experimenters had disappeared behind the curtain at which point the children would be told to begin. Upon hearing the signal to begin, all children stood, picked up their baskets, and hurried to the table to begin building their towers.

On the way to the table, the bottom fell out of one of the children’s baskets, scattering the blocks of that color. This was accomplished by remotely activating a device that caused the bottom of one basket to fall. The victim of the accident then had to collect the blocks from the floor, and transport them to the table without the use of the basket. In contrast, the bystander to the accident was unaffected by the accident and could continue to the table and begin building. Therefore, if the bystander chose to ignore the accident, the bystander would have finished the tower before the victim had even collected his/her blocks and brought them to the table. After both children had finished assembling their tower(s), one experimenter emerged from behind the curtain, handed both children large stickers, and thanked the children for their help.

### Coding

Based on Caplan and Hay’s (1989) coding scheme, the videotapes were coded for the presence of 4 empathic behaviors that the bystander could exhibit during the time interval from when the victim’s basket broke until the bystander placed his/her first block on the tower. After the bystander placed his/her first block, coding ceased. The first behavior consisted of the bystander looking at the victim for a total of at least 3 s. The second and third behaviors consisted of the bystander ceasing ongoing activity. Because the amount of time was not pre-defined by Caplan and Hay, we divided ceasing ongoing activity into two categories: (1) ceasing ongoing activity for 1 s or longer, which was defined as no longer walking towards the table, setting the basket down, removing a block from the basket, or placing a block on the tower and (2) ceasing ongoing activity until the victim placed a block on the tower which was the longest time that children ceased ongoing activity. Both amount of looking ≥ 3 s and ceasing ongoing activity for ≥ 1 s were coded by a female and male experimenter. The correlation between the two experimenters for amount of looking was r (53) = 0.934. Ratings were averaged for the final score yielding a spearman-brown coefficient of 0.97. Agreement on whether a bystander ceased ongoing activity for at least 1 s occurred for 52/55 pairs = 0.945. Disagreements were resolved by assuming the bystander had ceased ongoing activity.

The second type of ceasing ongoing activity required a significant delay for the bystander who had to wait until the victim collected the blocks that had scattered on the floor, transported them to the table, and placed one block on the tower, before the bystander placed his/her own block on the tower. The final behavior coded was whether the bystander actively intervened. Here we define active intervention as whether the bystander helped the victim by picking up the victim’s blocks or basket. Agreement was 100% for whether a bystander delayed placing a block on the tower until the victim placed one and for whether the bystander picked up one or more of the victim’s blocks or the victim’s basket.

Three of the measures- looking at victim for ≥ 3 s, ceasing ongoing activity until the victim placed his/her first block, and active intervention, were independent. That is, none required the other behaviors. Ceasing ongoing activity for ≥ 1 s however was necessary for ceasing activity until the victim placed his/her block and for active intervention.

## Data Availability

The dataset is available at figshare : https://doi.org/10.6084/m9.figshare.12894200.
